# Integrating Metabolomics and Network Pharmacology to Reveal the Mechanism of Thymoquinone Alleviating Renal Interstitial Fibrosis in UUO Mice

**DOI:** 10.3390/ijms27114901

**Published:** 2026-05-28

**Authors:** Yuanqing Liu, Huijing Zhang, Yanjun Dong, Gebin Li, Kai Fan, Zhihui Hao, Shuaiyu Wang

**Affiliations:** 1 College of Veterinary Medicine, China Agricultural University, Beijing 100193, China; lyq2220222@163.com (Y.L.);; 2 Innovation Centre of Chinese Veterinary Medicine, College of Veterinary Medicine, China Agricultural University, Beijing 100193, China; 3 Key Biology Laboratory of Chinese Veterinary Medicine, Ministry of Agriculture and Rural Affairs, Beijing 100193, China; 4 State Key Laboratory of Veterinary Public Health and Safety, College of Veterinary Medicine, China Agricultural University, Beijing 100193, China

**Keywords:** chronic kidney disease, renal interstitial fibrosis, thymoquinone, unilateral ureteral obstruction, metabolomics, network pharmacology, PI3K/AKT signaling pathway

## Abstract

Chronic kidney disease (CKD) represents a considerable health burden for both humans and veterinary patients globally. Renal fibrosis is the final common pathway for the progression of CKD to end-stage renal disease, which can eventually lead to renal failure. Thymoquinone (TQ), the primary bioactive constituent of *Nigella sativa*, has demonstrated significant antifibrotic potential; however, the specific molecular mechanisms underlying its renoprotective effects remain incompletely elucidated. This study aimed to investigate how TQ alleviates renal fibrosis to support its potential as a therapeutic agent. TQ’s renoprotective effects were evaluated in a murine unilateral ureteral obstruction (UUO) model using histopathology, Western blotting, immunofluorescence, and RT-qPCR. Network pharmacology and untargeted metabolomics were integrated to identify key pathways, which were further assessed through in vivo and in vitro experiments. TQ treatment attenuated UUO-induced renal interstitial injury. TQ treatment downregulated α-smooth muscle actin (α-SMA) and fibronectin, thereby suppressing myofibroblast activation and extracellular matrix (ECM) accumulation. Integrated multi-omics analyses indicated that the antifibrotic activity of TQ is associated with modulation of the PI3K/AKT signaling axis. Subsequent in vivo and in vitro studies suggested that TQ protects against renal injury by inhibiting aberrant PI3K/AKT signaling. This study found that TQ ameliorates renal interstitial fibrosis in UUO mice. The underlying mechanism appears to involve suppression of myofibroblast activation and ECM accumulation via inhibition of PI3K/AKT signaling. These findings highlight the therapeutic potential of TQ for renal fibrosis.

## 1. Introduction

Chronic kidney disease (CKD) is one of the most prevalent chronic diseases in clinical practice and represents a major public health challenge in both human and veterinary medicine. With a global median prevalence of approximately 9.5% in humans, CKD imposes a substantial burden on global health. In veterinary medicine, the overall prevalence of CKD is approximately 0.05% to 3.74% in dogs and ranges from 1% to 50% in cats, with markedly increased susceptibility in geriatric animals [1,2]. Clinically, CKD is characterized by progressive and irreversible deterioration of renal function accompanied by multisystemic complications, which severely compromise both the quality of life and survival of affected individuals. Despite its severity, effective therapeutic options remain limited; current management strategies are largely confined to symptomatic relief and renal replacement therapy. Consequently, the development of novel therapeutic interventions to arrest CKD progression is of paramount importance.

Renal fibrosis is universally acknowledged as the final common pathway in the progression of most CKD cases, manifesting primarily as glomerulosclerosis and renal interstitial fibrosis (RIF) [3]. It arises from maladaptive tissue repair, in which the uncontrolled accumulation of connective tissue ultimately results in structural scarring. From a pathological perspective, RIF is characterized by several key changes, including excessive accumulation of extracellular matrix (ECM), loss of tubular epithelial cells, fibroblast infiltration and activation, and decreased density of the peritubular capillary network [4]. Collagens I and III constitute the primary ECM components, and their aberrant deposition expands the interstitial space between the tubular basement membrane and capillaries, compromising renal architecture [5]. A central mechanism driving RIF is epithelial–mesenchymal transition (EMT), a process in which renal tubular epithelial cells acquire mesenchymal phenotype and functional traits of myofibroblasts [6]. During this transition, epithelial cells exhibit downregulation of adhesion proteins alongside a marked upregulation of myofibroblast markers, particularly α-smooth muscle actin (α-SMA) [7]. Transforming growth factor-β1 (TGF-β1) serves as a key profibrotic regulator of EMT and is widely recognized as a major driver of RIF progression [8]. TGF-β1 signaling induces the transdifferentiation of tubular epithelial cells into myofibroblasts, leading to loss of epithelial characteristics, suppression of adhesion molecules, and increased expression of fibronectin and α-SMA [9,10]. Additionally, fibrosis progression is closely associated with inflammatory cell infiltration and increased levels of cytokines, such as TNF-α, IL-6, and IL-1β, which collectively exacerbate RIF [11]. The PI3K/AKT pathway serves as a fundamental intracellular regulatory network, governing diverse biological processes including cell proliferation, metabolism, survival, and autophagy. While implicated in various pathologies ranging from cancer to metabolic disorders, accumulating evidence underscores the PI3K/AKT pathway as a key regulator of renal fibrosis [12,13]. Notably, its aberrant activation has been shown to trigger EMT in renal tubular epithelial cells.

Plant-derived natural compounds have shown broad renoprotective potential [14]. Thymoquinone (TQ), the primary bioactive constituent of *Nigella sativa*, has been reported to exert diverse pharmacological activities, including antioxidant, anti-inflammatory, antitumor, and antifibrotic effects [15]. Experimental evidence suggests that TQ confers renoprotection through multiple mechanisms, such as scavenging reactive oxygen species, enhancing antioxidant defenses, suppressing inflammatory mediators, inducing apoptosis, and inhibiting cellular migration [16,17]. Regarding its antifibrotic properties, TQ significantly downregulates TGF-β1 expression and downstream Smad2/3 phosphorylation, thereby suppressing myofibroblast activation and ECM deposition. Kou et al. further demonstrated that TQ inhibits the EMT in renal tubular epithelial cells by upregulating E-cadherin while downregulating mesenchymal markers, including Snail, ZEB1, and Vimentin [18]. In summary, although current literature indicates that the renoprotective actions of TQ are mediated predominantly through the TGF-β/Smad signaling pathway and inhibition of EMT, the precise molecular mechanisms underlying its effects in unilateral ureteral obstruction (UUO) and cellular models of RIF remain to be fully elucidated. Notably, TQ has been reported to exert significant antiproliferative and pro-apoptotic effects, and to reduce vascular endothelial growth factor (VEGF) expression in endothelial cells, via inhibition of the VEGFR2/PI3K/AKT signaling axis [19]. These observations suggest that TQ may alleviate renal fibrosis by suppressing fibroblast activation, attenuating angiogenesis, and limiting excessive ECM deposition. Collectively, these findings imply that the PI3K/AKT signaling pathway may represent a critical mechanism underlying the antifibrotic effects of TQ, although this hypothesis requires further experimental validation.

To address this gap, this study employed a UUO mouse model and a TGF-β1-induced NIH3T3 cell model to investigate the antifibrotic efficacy of TQ and the underlying mechanisms. Our results indicate that TQ effectively attenuates the progression of renal fibrosis in UUO mice. This protective effect was evidenced by significant alleviation of pathological injury and inflammation, as well as the inhibition of fibroblast activation and collagen deposition, was correlated with the modulation of the PI3K/AKT signaling pathway.

## 2. Results

### 2.1. TQ Alleviates Renal Pathological Injury in UUO Mice

**Figure 1:**
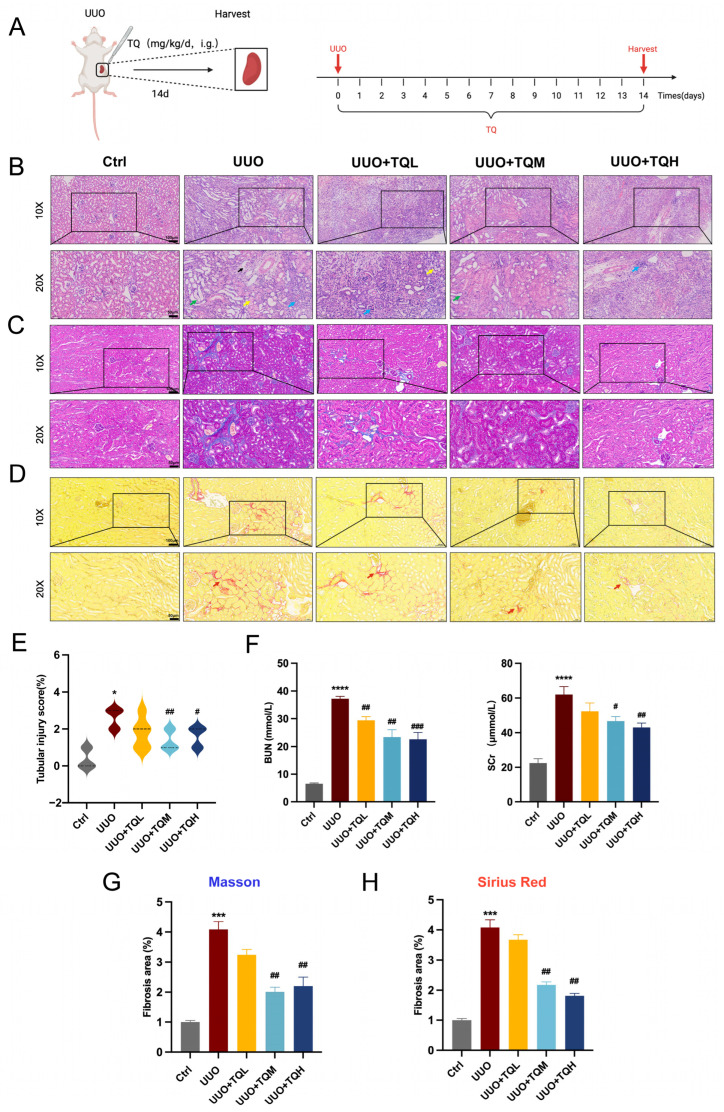
TQ alleviates renal pathological injury in UUO mice. (**A**) Experimental scheme of UUO and TQ treatment. (**B**) representative H&E-stained images of renal tissues (20×). In the UUO group, typical pathological changes were observed, including glomerular atrophy with dilated Bowman’s space (black arrows), tubular epithelial cell edema with extensive cytoplasmic vacuolation (yellow arrows), mild tubular dilation containing proteinaceous casts (green arrows), and diffuse inflammatory cell infiltration in the interstitium (blue arrows). a. Ctrl; b. UUO; c. UUO + TQL; d. UUO + TQM; e. UUO + TQH. The same group as below). (**C**) Representative morphological images of renal fibrosis stained with Masson’s trichrome (20×). (**D**) Representative morphological images of renal fibrosis stained with Sirius red staining (20×). Compared with the Control group, the TQM group shows mildly thickened interstitial collagen deposition (red arrows). (**E**) Assessment of renal tubular injury by H&E-staining. (**F**) Bar plots of BUN and SCr levels across experimental groups. (**G**) Quantitative analysis of interstitial collagen deposition based on Masson’s trichrome staining. (**H**) Quantitative analysis of interstitial collagen deposition based on Sirius Red staining. Statistical significance thresholds were established as follows: asterisks (*, ***, ****) indicate comparisons versus the Ctrl group (* *p* < 0.05, *** *p* < 0.001, **** *p* < 0.0001), whereas hash symbols (#, ##, ###) denote differences relative to the Model group (# *p* < 0.05, ## *p* < 0.01, ### *p* < 0.001).

After the UUO model was successfully established (Figure 1A), we performed an initial evaluation of the protective effect of TQ against RIF in mice. TQ administration significantly ameliorated UUO-induced pathological changes in a dose-dependent manner. Histopathological analysis based on H&E staining showed that, compared with the UUO group, the TQM and TQH groups exhibited reduced glomerular atrophy (black arrows), decreased vacuolar degeneration (yellow arrows), and attenuated renal tubular dilation (green arrows). Furthermore, interstitial inflammatory cell infiltration (blue arrows) was markedly reduced, and tissue disorganization was improved, as confirmed by the renal tubular injury scores (Figure 1B,E). Additionally, compared with the UUO group, serum biochemical analysis showed that BUN and SCr levels were markedly reduced in the TQM and TQH groups (Figure 1F). Masson staining results demonstrated a significant reduction in the area of blue-stained collagen fiber in both the TQM and TQH groups compared with the UUO group (*n* = 6, *p* < 0.01; Figure 1C,G). Similarly, Sirius Red staining showed a substantial decrease in the area of red-stained collagen fibers in the TQM and TQH groups (*p* < 0.01; Figure 1D,H). In summary, these results suggest that TQ effectively maintained renal structural integrity, suppressed pathological of extracellular matrix deposition, and delayed the progression of renal interstitial fibrosis, thereby conferring significant renoprotection.

### 2.2. TQ Alleviates Renal Fibrosis Progression

**Figure 2:**
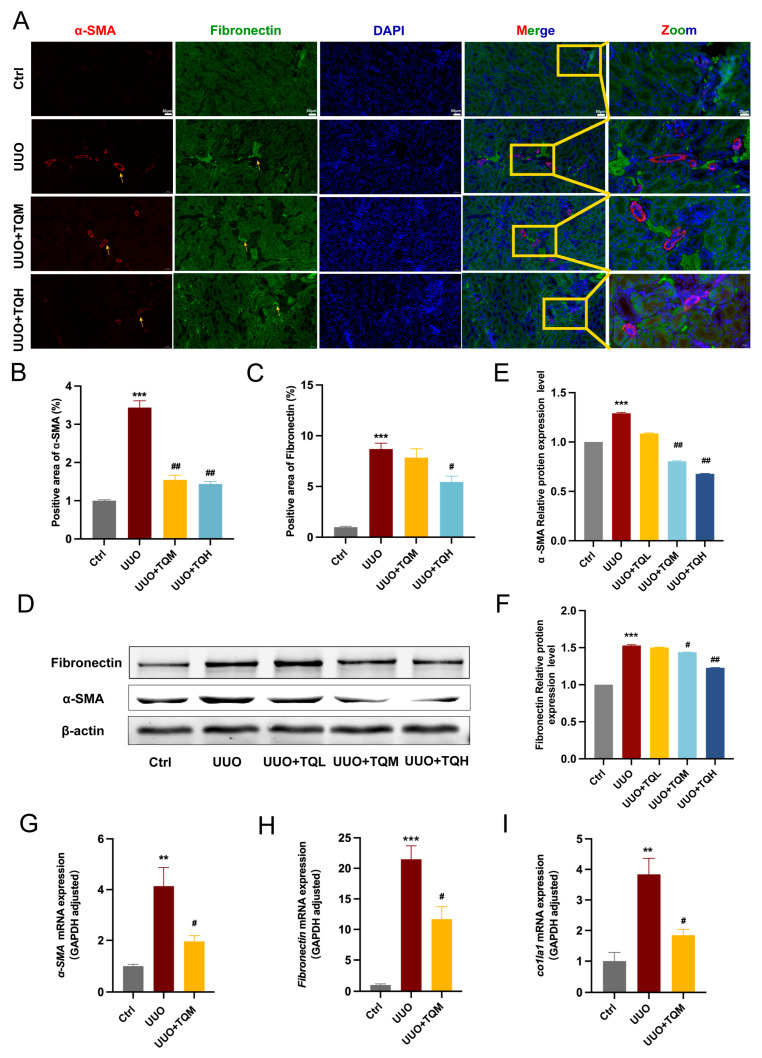
TQ alleviates renal fibrosis progression. (**A**) Representative immunofluorescence staining images of α-SMA and fibronectin in mouse renal tissues. Yellow arrows point to the positive expression areas of α-SMA and fibronectin. (**B**,**C**) Quantitative analysis of immunofluorescence intensity for α-SMA (**B**) and fibronectin (**C**). (**D**) Western blot analysis of α-SMA and fibronectin protein expression levels in mouse renal tissues. (**E**,**F**) Quantitative analysis of α-SMA (**E**) and fibronectin (**F**) protein expression in renal tissues. (**G**–**I**) Relative mRNA expression of *α-SMA* (**G**), *fibronectin* (**H**), and *Col1a1* (**I**) in renal tissues. Statistical significance thresholds were established as follows: asterisks (**, ***) indicate comparisons versus the Ctrl group (** *p* < 0.01, *** *p* < 0.001), whereas hash symbols (#, ##) denote differences relative to the Model group (# *p* < 0.05, ## *p* < 0.01).

To investigate how TQ exerts protective effects against renal fibrosis, we analyzed the expression of α-SMA and fibronectin using Western blot, RT-qPCR, and immunofluorescence staining. Immunofluorescence revealed that the fluorescence intensity of α-SMA was significantly decreased in the TQM and TQH groups compared with the UUO group (*p* < 0.01; Figure 2A,B). Further, the fluorescence intensity of fibronectin was markedly reduced in the TQH group (*p* < 0.05; Figure 2A,C). Western blotting results showed that α-SMA and fibronectin protein levels in kidney tissues were significantly reduced in the TQM and TQH groups as compared to the UUO group (*p* < 0.01; Figure 2D–F). In addition, RT-qPCR data indicated that mRNA expression levels of *α-SMA*, *fibronectin*, and *Col1a1* were significantly downregulated in the TQM group relative to the UUO group (*p* < 0.05; Figure 2G–I). Together, these results suggest that TQ mitigated RIF by suppressing myofibroblast activation and fibronectin expression.

### 2.3. Network Pharmacology Identifies the PI3K/AKT Signal Pathway Mediating the Therapeutic Effects of TQ

**Figure 3:**
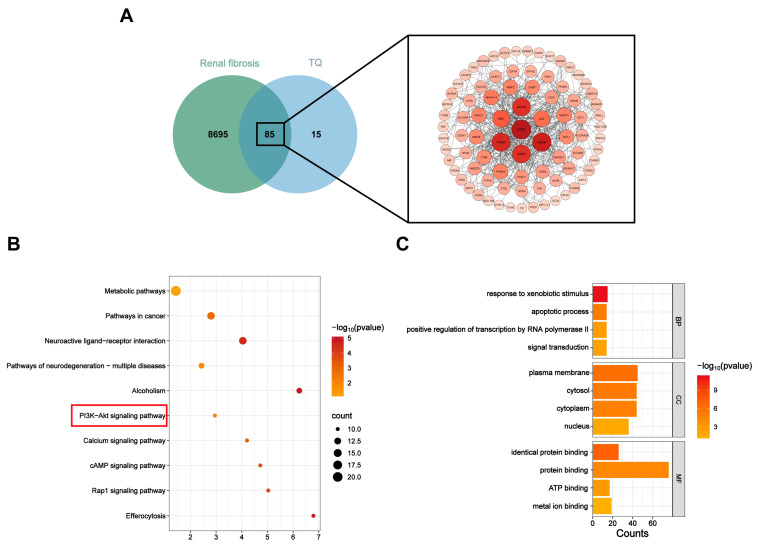
Network pharmacology analysis of TQ against renal fibrosis. (**A**) Venn diagram and PPI network of the overlapping therapeutic targets of TQ and renal fibrosis. (**B**,**C**) KEGG (**B**) and GO (**C**) enrichment analysis of overlapping targets.

By constructing a drug–target–disease interaction network, we identified 85 common targets of TQ and renal fibrosis. Based on these shared targets, a protein–protein interaction (PPI) network was constructed (Figure 3A). KEGG pathway analysis indicated that the antifibrotic effects of TQ are likely mediated through the coordinated regulation of multiple signaling pathways, including the PI3K/AKT, calcium, cAMP, and Rap1 pathways (Figure 3B). Furthermore, GO enrichment analysis revealed significant enrichment of these targets in diverse biological processes, including response to xenobiotic stimulus, apoptotic process, positive regulation of transcription by RNA polymerase II, and signal transduction. The enriched cellular components mainly included the plasma membrane, cytosol, cytoplasm, and nucleus, whereas the enriched molecular functions were primarily related to identical protein binding, protein binding, ATP binding, and metal ion binding (Figure 3C).

### 2.4. Metabolomics Revealed Key Differential Metabolites and Indicated the Crucial Role of the PI3K/AKT Signaling Pathway

**Figure 4:**
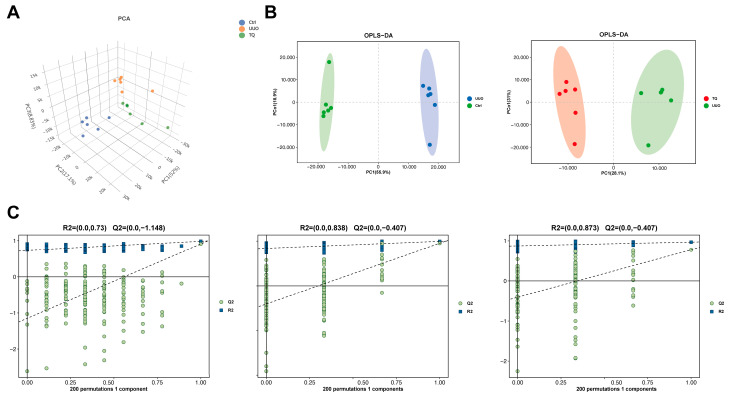
Validation of the metabolomics model. (**A**) 3D PCA score plot of kidney tissue samples from each group. (**B**) OPLS-DA score plot of kidney tissue samples from each group. (**C**) Permutation analysis of the OPLS-DA model: all samples (left), UUO vs. Ctrl (middle), TQ vs. UUO (right).

To further clarify the molecular regulatory mechanism by which TQ alleviates renal interstitial fibrosis in mice, kidney samples were collected from each group for metabolomic analysis. First, unsupervised principal component analysis (PCA) was performed to visualize sample distribution and assess data stability (Figure 4A). Subsequently, supervised orthogonal partial least squares discriminant analysis (OPLS-DA) was employed to identify metabolic differences among groups and screen for differential metabolites. The results demonstrated significant differences among the Ctrl, UUO, and TQM groups, with samples clustering tightly within each group (Figure 4B). Evaluation parameters R^2^X, R^2^Y, and Q^2^ indicated that the OPLS-DA model was robust without overfitting (Figure 4C), supporting its suitability for metabolite and pathway analysis.

**Figure 5:**
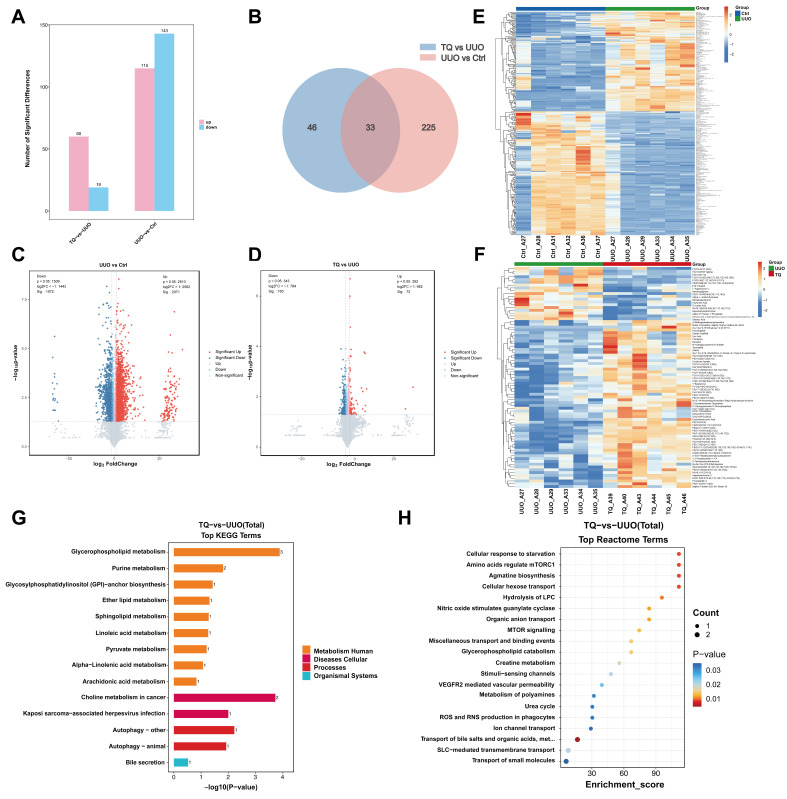
Metabolomic analysis of kidney tissue from UUO mice treated with TQ. (**A**) Summary of mass spectrometry data. (**B**) Venn diagram showing overlapping DMs between the TQ vs. UUO and UUO vs. Ctrl comparisons. (**C**,**D**) Volcano plots of DMs in the UUO vs. Ctrl (**C**) and TQ vs. UUO (**D**) comparisons. Red: upregulated DMs; blue: downregulated DMs. (**E**,**F**) Heatmaps of DMs in the UUO vs. Ctrl (**E**) and TQ vs. UUO (**F**) comparisons. (**G**) Bar chart of KEGG pathway enrichment. (**H**) Bubble plot of Reactome pathway enrichment analysis for TQ vs. UUO comparison.

According to the criteria for Differential metabolites (DMs) (*p* < 0.05, VIP > 1 and FC > 1), 258 DMs were identified in the UUO group compared with the Ctrl group, 115 metabolites were upregulated, and 143 were downregulated (Figure 5A,C,E). From the comparisons of TQM group with UUO group and UUO group with Ctrl group, a total of 33 overlapping differential metabolites were identified (Figure 5B). Compared with the UUO group, TQM treatment resulted in the upregulation of 60 metabolites and the downregulation of 19 metabolites (Figure 5A,D,F). KEGG pathway analysis indicated enrichment in several pathways, including Autophagy signaling, Glycosylphosphatidylinositol (GPI)-anchor biosynthesis, and Purine metabolism (Figure 5G,H).

**Figure 6:**
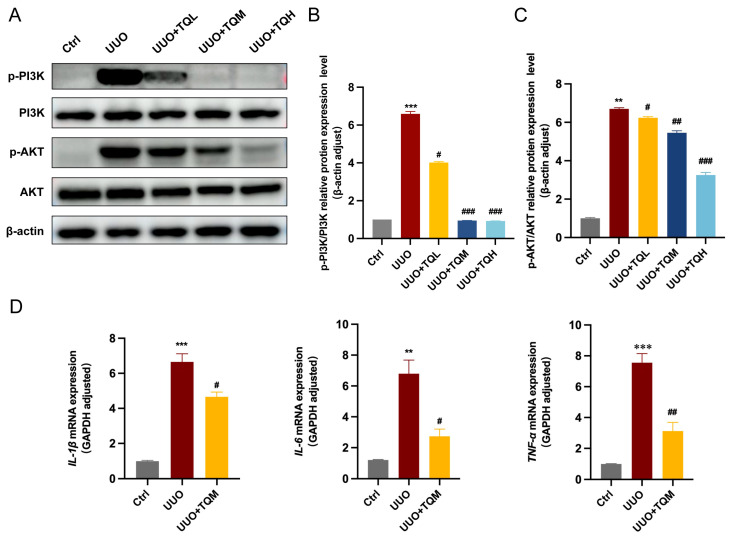
TQ exerts protective effects in UUO mice through modulation of the PI3K/AKT signaling pathway. (**A**) Western blot analysis of p-PI3K, PI3K, p-AKT, and AKT protein expression in mouse renal tissues. (**B**,**C**) Quantitative analysis of the p-PI3K/PI3K ratio (**B**) and p-AKT/AKT ratio (**C**). (**D**) Relative mRNA expression of *IL-1β*, *IL-6*, and *TNF-α* in mouse renal tissues. Statistical significance thresholds were established as follows: asterisks (**, ***) indicate comparisons versus the Ctrl group (** *p* < 0.01, *** *p* < 0.001), whereas hash symbols (#, ##, ###) denote differences relative to the Model group (# *p* < 0.05, ## *p* < 0.01, ### *p* < 0.001).

Based on these findings, we hypothesized that TQ exerts its renoprotective effect in UUO mice via the PI3K/AKT pathway and performed experimental validation. Western blot analysis results confirmed that the protein levels of p-PI3K and p-AKT were significantly elevated in the UUO group compared with the control group, indicating abnormal activation of the PI3K/AKT pathway following UUO surgery. The TQM groups showed significantly reduced protein levels of p-PI3K and p-AKT compared with the UUO group (Figure 6A–C). These results demonstrated that TQM effectively reversed abnormal activation of the PI3K/AKT signaling pathway in UUO mice. To further evaluate the anti-inflammatory effect of TQ, we measured the expression of key inflammatory cytokines in mouse kidney tissues by RT-qPCR. The results showed that TQM treatment significantly suppressed the UUO-induced increase in the mRNA expression of *IL-1β*, *IL-6*, and *TNF-α* (Figure 6D). These findings indicated that TQ effectively attenuated the inflammatory response and reduced the expression of pro-inflammatory cytokines in UUO mice.

### 2.5. TQ Suppresses TGF-β-Induced PI3K/AKT Signaling Pathway Activation

**Figure 7:**
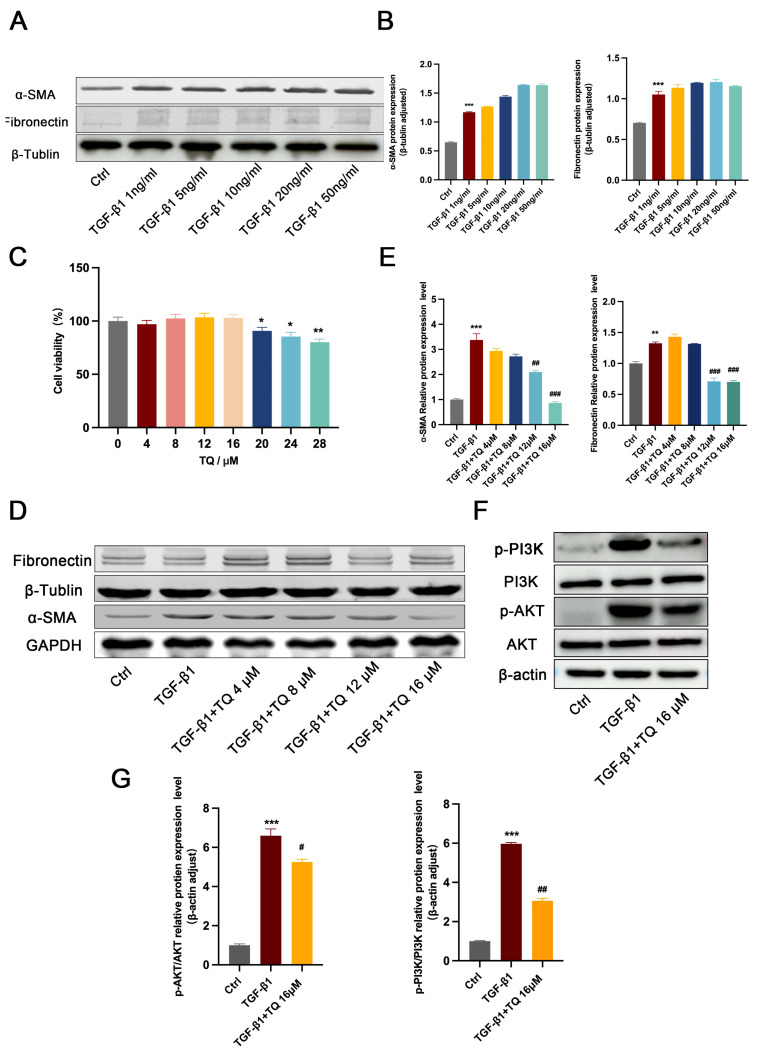
TQ suppresses TGF-β-induced PI3K/AKT signaling pathway activation. (**A**,**B**) Protein expression levels (**A**) and quantitative analysis (**B**) of α-SMA and fibronectin in NIH3T3 cells induced by different concentrations of TGF-β1. (**C**) C Evaluation of TQ cytotoxicity in NIH3T3 cells by CCK-8 assay. (**D**,**E**) Western blot analysis (**D**) and corresponding quantification (**E**) of α-SMA and fibronectin in NIH3T3 cells treated with different concentrations of TQ under TGF-β1 stimulation. (**F**,**G**) Western blot analysis (**F**) and quantitative analysis (**G**) of p-PI3K, PI3K, p-AKT, and AKT protein expression in NIH/3T3 cells treated with 16 μM TQ under TGF-β1 induction. Statistical significance thresholds were established as follows: asterisks (*, **, ***) indicate comparisons versus the Ctrl group (* *p* < 0.05, ** *p* < 0.01, *** *p* < 0.001), whereas hash symbols (#, ##, ###) denote differences relative to the Model group (# *p* < 0.05, ## *p* < 0.01, ### *p* < 0.001).

To further investigate the molecular mechanisms underlying TQ-mediated inhibition of RIF, we examined its effect on TGF-β1-induced proliferation and differentiation of NIH3T3 cells. Western blotting was performed to evaluate the optimal TGF-β1 concentration for model establishment. α-SMA protein expression increased in a concentration-dependent manner and peaked at 50 ng/mL. Fibronectin expression reached its maximum level at 10 ng/mL TGF-β1 (Figure 7A,B). Consequently, 10 ng/mL TGF-β1 was selected for subsequent experiments. CCK−8 assays revealed that TQ at concentrations below 20 μM exerted no significant effect on the viability of NIH3T3 cells. Cell viability was significantly reduced at 20 μM (*p* < 0.05). Thus, TQ at concentrations ranging from 0–20 μM was considered non-cytotoxic (Figure 7C).

The ability of TQ to inhibit TGF-β1-induced fibrosis in NIH3T3 cells was subsequently evaluated. Western blot results demonstrated that TGF-β1 significantly increased the expression of α-SMA and fibronectin (Figure 7D,E). In contrast, treatment with 12 μM and 16 μM TQ significantly reduced the expression of both proteins (*n* = 3, *p* < 0.01). To assess the effect of TQ on the PI3K/AKT pathway, the expression levels of p-PI3K, PI3K, p-AKT, and AKT were measured. TGF-β1 markedly increased p-PI3K and p-AKT levels, indicating activation of the PI3K/AKT signaling pathway. However, pretreatment with 16 μM TQ significantly decreased the protein levels of p-PI3K and p-AKT (*p* < 0.01, Figure 7F,G), suggesting that TQ inhibits activation of the PI3K/AKT pathway. These findings implied that TQ suppresses fibroblast activation through inhibition of the PI3K/AKT signaling pathway.

## 3. Discussion

RIF is widely recognized as the common pathological basis underlying the progression of CKD to end-stage renal failure. Its major pathological features include cellular injury, inflammatory responses, and excessive ECM deposition [20]. Renal tubular epithelial cells, which are highly vulnerable to metabolic, ischemic, and toxic insults, may lose their epithelial characteristics following barrier dysfunction and undergo EMT, thereby acquiring fibroblast-like properties [21]. This process is accompanied by increased expression of mesenchymal markers such as α-SMA and vimentin, which promotes myofibroblast formation and excessive ECM deposition [7]. A Because α-SMA is widely used as a marker of myofibroblast activation and fibronectin is a major ECM component driving fibrosis, their marked downregulation in our study provides direct evidence that TQ suppresses myofibroblast activation and limits ECM accumulation. Meanwhile, persistent inflammatory cells infiltration and excessive release of cytokines, including *TNF-α*, *IL-6*, and *IL-1β* further activate fibroblasts, amplify local inflammation, and disrupt microvascular homeostasis [22]. Inflammatory responses and EMT-related changes induced by tubular epithelial injury interact to promote continuous ECM accumulation and progressive epithelial damage, ultimately establishing a self-amplifying fibrotic process.

**Figure 8:**
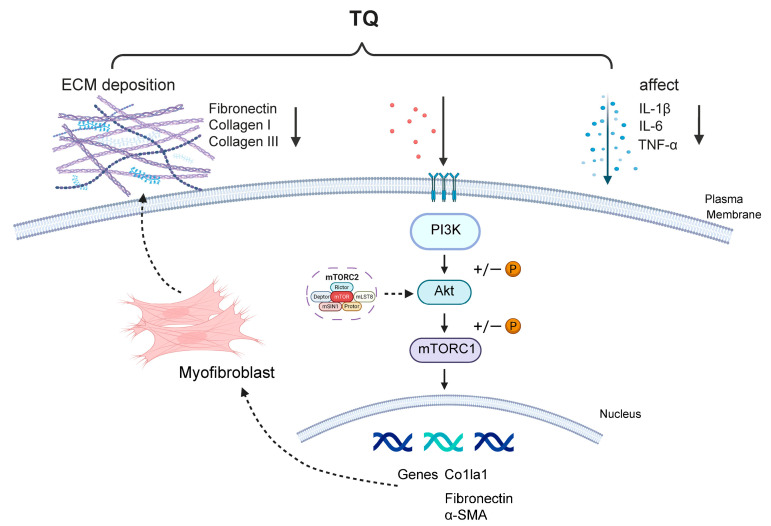
Schematic diagram of the proposed renoprotective mechanism of TQ. As illustrated in the diagram, TQ may exert its renoprotective effects through two main mechanisms. Primarily, it inhibits the phosphorylation of AKT within the PI3K/AKT signaling pathway. This inhibition leads to the downregulation of gene expression for *Col1a1*, *α-SMA*, and *fibronectin*, thereby suppressing fibroblast activation and reducing ECM collagen deposition. Concurrently, TQ downregulates the expression of pro-inflammatory cytokines, thereby exerting anti-inflammatory effects. Together, these actions contribute to the renoprotective effects of TQ.

In the present study, the marked attenuation of glomerular atrophy and tubular dilatation indicates that TQ effectively preserves renal structural integrity. Our further investigations suggested that TQ alleviates the pathological deposition of the ECM during renal fibrosis by modulating the phosphorylation cascade of the PI3K/AKT signaling pathway (Figure 8). Collectively, these findings suggest that TQ preserves both the structural and functional integrity of the kidney, thereby limiting the progression from acute parenchymal injury to chronic interstitial fibrosis. This study provides further mechanistic evidence supporting the PI3K/AKT pathway as a potential therapeutic target for renal fibrosis.

The development of RIF is closely associated with the aberrant activation of multiple signaling pathways, among which the PI3K/AKT pathway is recognized as a key regulator of cell proliferation, survival, metabolism, and inflammatory reaction [23]. This finding is consistent with previous observations by Huang et al., who reported that TQ inhibited PI3K/AKT signaling in hepatic fibrosis models, suggesting that the potentially broad protective role of TQ in fibrosis across multiple organs [24].

Unlike conventional single-target natural compounds such as hesperidin, TQ has been demonstrated to possess multi-pathway regulatory capacity, influencing key processes including inflammation, oxidative stress, and cell proliferation [25]. This characteristic may enhance its antifibrotic efficacy and highlight the therapeutic potential of natural product-based strategies, in which multi-target and multi-mechanistic modulation may improve treatment outcomes. Notably, TQ may suppress fibroblast activation by modulating the crosstalk between inflammatory responses and profibrotic signaling pathways. This mechanism complements existing studies on the roles of TGF-β/Smad and Wnt/β-catenin pathways in renal fibrosis, suggesting that multiple fibrosis-related pathways can be modulated simultaneously by TQ. Further investigation of TQ’s synergistic effects across different pathways, along with assessment of its long-term safety, is critical for translating natural product-based antifibrotic therapies into clinical applications.

Network pharmacology was employed to predict the molecular mechanisms underlying the therapeutic effects of TQ against RIF. Meanwhile, metabolomic analysis suggested that the antifibrotic effects of TQ in mice were associated mainly with the regulation of purine metabolism, amino acid metabolism, and related metabolic pathways. Previous studies have shown that the PI3K/AKT-mTORC1 axis coordinates multiple metabolic processes, including glucose, lipid, and nucleotide metabolism, to meet the energetic and biosynthetic demands of cell growth [26]. Notably, the selection of the PI3K/AKT signaling pathway for further validation was based on an integrated interpretation of our multi-omics data. Specifically, network pharmacology identified PI3K/AKT signaling as a major enriched pathway potentially involved in the antifibrotic action of TQ, whereas untargeted metabolomics independently revealed significant alterations in downstream metabolic processes, particularly autophagy and purine metabolism. Given that the PI3K/AKT pathway is well-established in the literature as a master upstream regulator of both autophagy and nucleotide metabolism [27,28], these findings synergistically guided our focus toward this pathway for subsequent experimental validation. Consistent with this inference, our molecular experiments further demonstrated that TQ modulated PI3K/AKT signaling, thereby inhibiting myofibroblast activation and excessive ECM deposition and ultimately alleviating renal structural injury. Together, these findings provide mechanistic insight into the antifibrotic effects of TQ and highlight PI3K/AKT signaling as a potential therapeutic target in renal fibrosis.

## 4. Materials and Methods

### 4.1. TQ Preparation

TQ (HY-D0803) was purchased from MCE (Monmouth Junction, NJ, USA). For the in vivo study, TQ was dissolved in corn oil to prepare dosing formulations for administration at 5, 10, and 15 mg/kg. The formulations were sonicated and vortexed to ensure homogeneity and were stored at 4 °C before use. For in vitro assays, TQ was dissolved in DMSO to prepare stock solutions, which were stored at −20 °C until use.

### 4.2. Animals Grouping and Treatment

Thirty male C57BL/6N mice (8–10 weeks old; specific pathogen-free [SPF]) were obtained from Vital River Co., Ltd. (Beijing, China). The mice were housed under SPF conditions at 25 ± 1 °C, with ad libitum access to food and water. All animal experiments were approved by the Animal Ethics Committee of China Agricultural University (Approval No. Aw11214202-2-02).

Mice were randomized into 5 groups (*n* = 6 per group) after 1-week acclimatization. With the exception of the control group (Ctrl), all mice were subjected to UUO surgery according to the protocol described by previous study [27]. Following the establishment of the model, mice were divided into treatment groups: (1) UUO model group (UUO); (2) UUO + low-dose TQ (TQL, 5 mg/kg); (3) UUO + medium-dose TQ (TQM, 10 mg/kg); and (4) UUO + high-dose TQ (TQH, 15 mg/kg).

They were orally administered once daily for 14 consecutive days. Mice in the TQ treatment groups received TQ dissolved in corn oil, while mice in the Ctrl and UUO groups received an equivalent volume of corn oil (vehicle). On day 14 post-surgery, all mice were euthanized via intraperitoneal injection of sodium pentobarbital. Kidney tissues were subsequently collected, rinsed, and processed for further analysis.

### 4.3. Histopathological Observations

Fresh renal tissues were harvested, fixed in 4% paraformaldehyde for 32 h, and then processed for dehydration and paraffin embedding. To assess renal injury and fibrosis, sections were subjected to Hematoxylin and Eosin (H&E) staining for histopathological evaluation, as well as Masson’s trichrome and Sirius Red staining to visualize collagen deposition. The remaining renal tissue was immediately stored at −80 °C.

### 4.4. Tubular Injury Score

Renal tubular injury was evaluated on H&E-stained sections following the method described by previous study [28]. Ten randomly selected non-overlapping fields (×200 magnification) were examined for each sample, ensuring equal representation of the renal cortex and corticomedullary junction. Tubular injury severity was scored semi-quantitatively from 0 to 5 according to the proportion of damaged tissue: 0 (normal), 1 (<10%), 2 (10–25%), 3 (26–50%), 4 (51–75%), and 5 (>75%). To minimize bias, the analysis was performed blindly by two independent investigators.

### 4.5. Blood Urea Nitrogen (BUN) and Serum Creatinine (SCr) Levels Measurement

Serum was obtained by collecting blood samples, incubating them at room temperature for 30 min, and centrifuging at 3000 rpm for another 30 min. The levels of BUN and SCr were quantified using commercial assay kits (Mlbio, Shanghai, China). Optical density (OD) values at 340 nm and 546 nm were measured separately using a microplate reader.

### 4.6. Immunofluorescence Staining

Kidney tissue sections were subjected to antigen retrieval with EDTA retrieval solution and subsequently blocked at room temperature for 20 min. Sections were incubated with primary antibodies overnight at 4 °C, then washed with PBS. Then, the sections were incubated with fluorescently labeled secondary antibodies. Images were acquired using a Nikon E100 fluorescence microscope (Nikon, Tokyo, Japan) and analyzed with Nikon NIS-Elements Br 3.0 software.

### 4.7. Real-Time Quantitative PCR Assay

Total RNA was extracted from renal tissues or cells under RNase-free conditions with a commercial RNA extraction kit, and its concentration and purity were determined with a NanoDrop 3000 spectrophotometer (Thermo Fisher Scientific, Waltham, MA, USA). Subsequently, RT-qPCR was listed in a Roche LightCycler 480 Real-Time PCR System (Roche Diagnostics, Basel, Switzerland) in strict accordance with the manufacturer’s instructions. The reaction program and primer sequences are provided in the Supplementary Table S1. For normalization, *GAPDH* was used as the internal reference gene, and the 2^−ΔΔCT^ method was applied to calculate relative gene expression levels.

### 4.8. Western Blotting

Kidney tissue stored at −80 °C was lysed in lysis buffer to extract total protein, then quantified protein concentrations using a commercial BCA kit. After electrophoresis and transfer, the PVDF membrane was blocked with 5% non-fat milk for 1 h, washed three times with TBST, incubated with primary antibody overnight, incubated with the corresponding secondary antibody for 1 h, and visualized. The antibodies used are listed in Supplementary Table S2. Fluorescent signals were detected at 680 nm and 800 nm using a Sapphire Biomolecular Imager (Azure Biosystems, Dublin, CA, USA).

### 4.9. Network Pharmacology Analysis of the Mechanisms and Pathways of TQ in Intervening Renal Fibrosis

Potential protein targets of TQ were identified via the SWISSTargetPrediction database (http://swisstargetprediction.ch/). Disease targets associated with renal fibrosis were obtained from the Genecards (https://www.genecards.org/), OMIM (https://www.omim.org/), and TTD (https://ttd.idrblab.cn/) databases using the keyword “renal fibrosis”. After integration and deduplication, the targets were combined into a unified target set.

Overlapping targets between TQ and renal fibrosis were identified via a Venn diagram, which were considered potential targets mediating the antifibrotic effects of TQ. A “TQ–target–renal fibrosis” interaction network was subsequently generated using Cytoscape 3.7.1 to visualize the multi-target mechanisms of action. protein–protein (PPI) interaction analysis was performed by importing the overlapping targets into the STRING database (https://cn.string-db.org/), with “Homo sapiens” designated as the restricted species. The PPI data were subsequently visualized as a network using Cytoscape 3.7.1.

The overlapping targets were subjected to Gene Ontology (GO) functional annotation and Kyoto Encyclopedia of Genes and Genomes (KEGG) pathway enrichment analyses via the DAVID database (https://davidbioinformatics.nih.gov/). Homo sapiens was designated as the study species, and official gene symbols were employed for annotation in the analysis. The top 4 significantly enriched GO terms—covering biological processes, molecular functions, and cellular components and the top 10 KEGG pathways, ranked by *p*-value, were selected for results interpretation and visualization.

### 4.10. Metabolomic Analysis

Metabolomics sample analysis was performed on an LC–MS platform comprising a ACQUITY UPLC I-Class Plus (Waters, Milford, MA, USA) system and a Q Exactive HF high-resolution mass spectrometer (Thermo Fisher Scientific, Waltham, MA, USA), with an ACQUITY UPLC HSS T3 column (100 mm × 2.1 mm, 1.8 μm) employed for chromatographic separation. We set the column temperature at 45 °C, the flow rate at 0.35 mL/min, and the injection volume at 5 μL. The mobile phases included two components: (A) water containing 0.1% formic acid and (B) acetonitrile. The gradient elution conditions: 5% B (0–2 min), increasing to 30% B (2–4 min), 50% B (4–8 min), 80% B (8–10 min), 100% B (10–15 min), and returning to 5% B (15–16 min). Mass spectrometry was carried out using positive and negative electrospray ionization modes, with spray voltages of 3800 V and −3000 V for the positive and negative modes. The auxiliary gas heater temperature was set at 350 °C, and the capillary at 320 °C.

### 4.11. Cell Culture, Treatment

NIH3T3 cells were purchased from Wuhan Procell Life Science & Technology Co., Ltd. (CL-0171, Wuhan, China). Cells were maintained in high-glucose DMEM (Gibco, Grand Island, NY, USA) supplemented with 10% calf serum (CS; Sigma-Aldrich, St. Louis, MO, USA, B7446) under standard culture conditions (37 °C, 5% CO_2_, and saturated humidity). Cells were evenly seeded into 6-well plates and subjected to serum starvation for 10 h after attachment to synchronize the cell cycle prior to subsequent experiments.

For the cytotoxicity assay, NIH3T3 cells were exposed to doses of 0, 4, 8, 12, 16, 20, 24 and 28 μM and maintained for 24 h. After exposure, each well was added with 10 μL of the CCK-8 reagent (RXH655-1, Ruixin Biotech, Quanzhou, China) and subjected to a 2 h incubation at 37 °C. OD at 450 nm was quantified on a microplate reader for the assessment of cell viability.

For the model induction assay, NIH3T3 cells treated with transforming growth factor-β1 (TGF-β1; 1, 5, 10, 25, 50, and 100 ng/mL) (100-21-10UG, PeproTech, Cranbury, NJ, USA) at multiple distinct concentrations for 24 h to establish the fibrotic model.

For the drug intervention assay, NIH3T3 cells were co-treated with TQ (4, 8, 12, 16 μM) and TGF-β1 (10 ng/mL) for 24 h to assess the modulatory effects of TQ on TGF-β1-induced responses.

### 4.12. Statistical Analysis

Data are expressed as the mean ± standard error of the mean (SEM). Statistical analyses and data visualization were performed using GraphPad Prism 9.0 software (GraphPad Software, San Diego, CA, USA). Student’s t-test was used for comparisons between two groups, while one-way ANOVA with Tukey’s post hoc test was applied for multiple group comparisons. The quantification of relative protein grayscale values was conducted using ImageJ 13.0.6 software. We defined statistical significance as * *p* < 0.05 and ** *p* < 0.01 when compared with the Ctrl group; # *p* < 0.05 and ## *p* < 0.01 when compared with the Model group.

## 5. Conclusions

In this study, TQ, a natural bioactive compound, was identified as a potentially effective antifibrotic agent against renal fibrosis. TQ markedly ameliorated renal interstitial fibrosis in mice with UUO-induced renal injury, as evidenced by reduced inflammatory infiltration and decreased ECM deposition. Both in vivo and in vitro experiments indicated that TQ effectively suppressed myofibroblast activation, accompanied by downregulation of the profibrotic proteins α-SMA and fibronectin. Network pharmacology analysis and molecular assays further suggested that the renoprotective effects of TQ were closely associated with suppression of PI3K/AKT signaling, as reflected by downregulation of fibrosis-related genes and reduced phosphorylation of key signaling proteins. Collectively, these results indicate that TQ attenuates renal fibrosis, through modulation of the PI3K/AKT pathway, supporting its potential as a novel antifibrotic agent.

## Data Availability

The original contributions presented in this study are included in the article/Supplementary Materials. Further inquiries can be directed to the corresponding author.

## Supplementary Materials

The following supporting information can be downloaded at: https://www.mdpi.com/article/10.3390/ijms27114901/s1.

## Abbreviations

BUN: Blood urea nitrogen; CKD, Chronic kidney disease; ECM, Extracellular matrix; EMT, Epithelial–mesenchymal transition; GO, Gene ontology; H&E, Hematoxylin and eosin; KEGG, Kyoto Encyclopedia of Genes and Genomes; OPLS-DA, Orthogonal partial least squares discriminant analysis; PCA, Principal component analysis; PPI, Protein–protein interaction; RIF, Renal interstitial fibrosis; SCr, Serum creatinine; TGF-β1, Transforming growth factor-β1; TQ, Thymoquinone; UUO, Unilateral ureteral obstruction; α-SMA, α-smooth muscle actin.
