# Supplementary material for: Integrating Metabolomics and Network Pharmacology to Reveal the Mechanism of Thymoquinone Alleviating Renal Interstitial Fibrosis in UUO Mice
Source: Int J Mol Sci. 2026 May 28;27(11):4901. doi: 10.3390/ijms27114901 (PMC13257271; doi:10.3390/ijms27114901)
Supplement: Supplementary file 1 [file ijms-27-04901-s001.zip › ijms-4257222-supplementary.pdf]

1. The reaction program and all primer information in Real-time quantitative PCR assay.

The quantitative PCR amplification was performed in a total reaction volume of 20 μL. The thermal cycling protocol was as follows: initial denaturation at 95°C for 10 minutes; followed by 40 cycles of denaturation at 95°C for 15 seconds and annealing/extension at 60°C for 1 minute. A dissociation curve analysis was carried out after amplification, with the temperature rising gradually from 60°C to 95°C.

Table S1. Details of all primer sequences used in this study.

Genes	Species	Sequence (5′ to 3′)
α-SMA	Mouse	Forward GGCACCACTGAACCCTAAGG<br>Reverse ACAATACCAGTTGTACGTCCAGA
Fibronectin	Mouse	Forward TACCAAGGTCAATCCACACCCC<br>Reverse CAGATGGCAAAAGAAAGCAGAGG
Col1a1	Mouse	Forward TGTTGGTCCTGCTGGCAAGAATG<br>Reverse GTCACCTTGTTCGCCTGTCTCAC
IL-6	Mouse	Forward AAGCCAGAGCTGTGCAGATGAG<br>Reverse TGGCATTTGTGGTTGGGTCAG
IL-1β	Mouse	Forward ATGATGGCTTATTACAGTGGCAA<br>Reverse ACAAAGCGTTTTCCGCTTCTT
TNF-α	Mouse	Forward CCTCTCTCTAATCAGCCCTCTG<br>Reverse GAGGACCTGGGAGTAGATGAGGT
GAPDH	Mouse	Forward ACATCATCCCTGCCTCTACTGG<br>Reverse AGTGGGTGTCGCTGTTGAAGTC

2. Table S2. Details of all antibodies used in this study.

Antibody	Catalog number	Brand	Dilution
Fibronectin	ab2413	Abcam	1:1000
a-SMA	19245S	CST	1:1000
p-PI3K	4228S	CST	1:1000
PI3K	4292S	CST	1:1000
p-AKT	9271S	CST	1:1000
AKT	C67E7	CST	1:1000
β-Actin	TA-08	ZSGB-BIO	1:2000
β- Tubulin	TA-10	ZSGB-BIO	1:2000
GAPDH	Ab181602	Abcam	1:10000
